# The Medical Society of the State of New York

**Published:** 1897-03

**Authors:** 


### THE MEDICAL SOCIETY OF THE STATE OF NEW YORK.

THE ninety-first annual meeting of the Medical Society of the State of New York, held at Albany, January 26, 27 and 28, 1897, under the presidency of Dr. James D. Spencer, of Watertown, will always be regarded as one of the most excellent meetings of this society, noted though it is for the good quality of its annual sessions. In the first place, the attendance was large and representative ; in the second, the papers and discussions were well up to the high standard heretofore set by the society ; and in the third, the whole proceedings were marked by a smoothness and freedom from friction that indicated an experienced hand at the helm.

These meetings of late years have been ushered in with a preliminary social function called the ex-president's annual dinner, and this year these dignitaries were entertained at the Fort Orange Club, the usual place, by Dr. F. C. Curtis, secretary of the society. This proved one of the most pleasant and entertaining occasions of its kind. The host displayed an aptitude for preventing monotony as well as for inspiriting the occasion with timely remarks that stimulated a flow of wit and eloquence.

The president's inaugural address was a paper of comprehensive dignity and showed a knowledge of the status of the medical profession, not only within the limits of this commonwealth, but all over the Union. His suggestion that a national confederation of committees of legislation of state medical societies, should be organised, to meet annually at Washington, points to the practical solution of an important question. Medical legislation that has its origin outside of medicine is not likely to conduce to its advancement ; it should always originate within the ranks of the profession after due consideration and careful preparation by representative physicians.

The committee of legislation of this society is always one of importance. Dr. A. Walter Suiter, chairman, presented a clear report of the workings of this committee during the past year showing that it had been active in opposing unwise legislation as well as in promoting measures of professional importance.

The committee on hygiene, Dr. Henry R. Hopkins, chairman, made a full report, in which it was advised that local societies for the promotion of public health should be organised in every county in the state and that reports should be made to this committee. In this way it is expected that an intelligent public sentiment will be created tending to educate the people concerning the essential rules of hygiene and sanitation—measures of importance in the prevention of disease. Buffalo, under stimulus of this suggestion, has taken the initiative in this matter, as will be noted by reference to our leading article in this issue.

The report of the state board of medical examiners attracted attention, and is published in full elsewhere in this edition. A careful reading of it will show the value of the work that is being done in preventing unqualified doctors from practising in this state, and indicates the conscientious performance of an onerous duty by the board.

The scientific work of the society has been epitomised already in the weekly journals and will be published in full in the transactions. An obstetric discussion, in which five or six men of prominence participated, was a fitting termination to the afternoon session of the first day. The discussion on the relation of the drinking of impure water to disease is calculated to yield beneficial results. Dr. T. B. Carpenter, of Buffalo, distinguished himself in an able paper on the methods of the purification of impure water. The discussion on diseases of the rectum, led by Prof. Joseph M. Mathews, of Louisville, Ky., marked a distinctive advance in this line of study. Dr. Mathews is a man of science, an easy and forceful speaker, and presents his points in a logical manner that carries conviction.

The president's annual address dealt with the subject of The country doctor, and was interestingly handled by a man of experience. It was well received by the society and commanded a vote of thanks at its close.

The annual dinner at the Kenmore concluded the functions of the second day. It was well attended by representative physicians and guests of prominence. Dr. J. M. Mathews made a capital speech, in which he again demonstrated his oratorical powers with a vocabulary of elegant diction. He received congratulations on all sides for his eloquent and manly stand with reference to medical educational problems that are just now absorbing so much attention. His peroration was an appropriate anti-climax to the remarks of Mr. William Barnes, Jr., that were so out of place.

At the Thursday morning session the scientific work was finished and the society adjourned.

---

The following officers for the ensuing year were elected : President, Seneca D. Powell, of New York ; vice-president, Lucien Howe, of Buffalo ; secretary, F. C. Curtis, of Albany ; treasurer, C. H. Porter, of Albany. Chairmen of committees—Arrangements, W. J. Nellis, of Albany ; by-laws, H. D. Wey, of Elmira ; hygiene, Henry R. Hopkins, of Buffalo ; legislation, A. Walter Suiter, of Herkimer ; ethics, J. M. Winfield, of Brooklyn ; prize essays, E. D. Fisher, of New York ; publication, F. C. Curtis, of Albany.

